# Supplementary material for: Are carbon emissions trading and green financial instruments synergistic? -Comprehensive quantitative research based on content analysis
Source: PLoS One. 2024 Mar 7;19(3):e0298601. doi: 10.1371/journal.pone.0298601 (PMC10919601; doi:10.1371/journal.pone.0298601)
Supplement: S1 Data — (ZIP) [file pone.0298601.s001.zip › Data/地级市Z_F工作报告.zip!/─■╧─/╓╨╬└╩╨/└┤╫╘╣1⁄2╓┌║┼í╛┬φ┐╦╩2╛▌═°í┐_2016.docx]

各位代表：

现在，我代表市人民政府，向大会作工作报告，请予审议，并请政协委员和列席人员提出意见。

2015年及“十二五”工作回顾

2015年是中卫设市以来面临困难最多的一年，我们认真贯彻落实中央、自治区和市委的各项决策部署，坚持以“1846”工作计划为统领，以“三基本”为重要抓手，以提高发展质量和效益为中心，攻坚克难，开拓创新，主动作为，统筹稳增长、调结构、优环境、惠民生、促改革、提效能各项工作，经济社会总体呈现稳中有进、稳中向好态势。全市实现地区生产总值316.6亿元，增长6.1%；全社会固定资产投资351.6亿元，增长5.3%；地方一般公共预算收入21.3亿元，增长9.4%；一般公共预算支出118.8亿元，增长11.9%；社会消费品零售总额60.6亿元，增长6.9%；城镇和农村常住居民人均可支配收入分别达21604元和8002元，增长8.4%和8.1%。事非经过不知难。这是在积极应对经济持续下行压力，扎实进行腾格里沙漠环保问题整治、土地问题整改等情况下取得的，难能可贵，来之不易。

一年来，我们重点抓了六方面工作：

一、坚持精准施策，经济运行企稳回升

狠抓项目建设。建立市级领导 “3个10”包抓企业责任制和“8+2”重点项目推进机制，年初383个计划项目和40个重点项目全部开工建设。国开行、农发行重点建设基金支持4批41个项目18亿元，解决了部分项目资金短缺问题。11家企业纳入券商新三板挂牌辅导程序。云计算、旅游等6个招商组围绕重大节会、重点地区、重点企业不间断招商，全年实施区外招商引资项目82个，到位资金260亿元。

狠抓政策落实。不折不扣落实自治区新“工业18条”，出台“中卫市工业保增长26条”，探索建立财政支持地方经济发展转贷过桥、助保贷和财政支农资金贴息等模式，设立“四主一化”产业转型专项基金，落实差别化电价、电力直接交易、过路过桥费减免等优惠政策，支持和帮助企业降成本、渡难关。32家骨干企业完成电力直接交易量64.8亿度，为铁合金、电石企业给予每千瓦时用电0.03元补贴。依法为企业办理减免（退）税4.6亿元。

狠抓协调服务。实施停产半停产企业脱困计划，帮助兴尔泰化工等4家企业开足马力，新华实业等6家企业恢复生产，复产率达45%以上。因企施策稳存量、促增量、控减量，宁夏协鑫N型单晶硅等12个项目开工建设，天元锰业三期30万吨电解锰等10个项目投产达效，新增产值31亿元。天元锰业与香港百灵达等3家企业合作上市，引入战略投资177亿元。实现规模以上工业增加值91.9亿元，增速由2月份-4.9%回升至全年6.9%，腾格里沙漠环保问题倒逼发展绿色化，地区生产总值增速由一季度0.5%逐季回升到全年6.1%。

二、主动调整结构，产业转型加快推进

旅游产业实现新突破。实施旅游新镇、沙坡头景区提升改造、沙坡头水镇等重点项目28个。旅游新镇主体工程竣工，腾格里湿地金沙岛被命名为首批自治区级旅游度假区。成功举办大漠黄河国际旅游节、环青海湖国际公路自行车赛、2015国际轮滑公开赛等旅游节会赛事活动，中卫的知名度和影响力不断提升。全年接待游客430万人次，旅游总收入33亿元，分别增长26%和25%，第三产业增加值增长6.4%。

信息产业迈出新步伐。解决了网络带宽和电价等云计算发展瓶颈问题。美国亚马逊AWS、云创公司2个新一代超大型数据中心即将投用，中关村科技产业园中卫云中心建成运行，西部云基地公司等16家企业入驻，一批云制造、云服务、云应用项目陆续落户、开工。环保、交通、卫生、旅游、媒体等云应用项目加快推进。实施“互联网+”行动计划，建成100家农村电商服务站，全市从事云计算及配套产业的法人企业达125家，仅2015年新增注册67家。中卫列入“宽带中国”示范城市和全国智慧城市。

交通物流开创新局面。海原至海兴开发区快速通道即将通车，海原至同心高速、吴忠至中卫城际铁路开工建设，干武铁路增建二线工程加快推进，包兰铁路中卫至兰州段扩能改造、乌玛高速中卫段开展前期工作。中国物流中卫物流园加快建设，中宁星火国际物流园智能化信息中心投入运营。新建农村公路269.4公里。优化公交线路6条，沙坡头区新建候车亭268座、港湾式站台10处20个，海原城乡客运一体化项目完工。

特色农业取得新成效。实施枸杞、硒砂瓜品质品牌保护和养殖业节本增效工程，形成卫宁36万亩枸杞、环香山90万亩硒砂瓜、香海山区清真牛羊肉和“16万亩设施+24万亩露地”蔬菜四大特色优势产业板块，培育壮大农产品加工龙头企业118家。粮食生产获“十二连丰”。香山硒砂瓜、健商堂枸杞、南月马铃薯入选2015年“全国百家合作社百个农产品品牌”。建成中宁电子商务孵化园，探索“互联网+特色农产品”营销新模式，枸杞销售收入增长22.7%。

工业发展呈现新格局。大力发展科技含量高、技术支撑力强的战略性新兴产业，以电解金属锰为代表的冶金产业、以蛋氨酸为代表的精细化工业、以N型单晶硅为代表的新材料产业、以枸杞产品系列开发为代表的农产品加工业、以光伏风电为代表的新能源产业稳步发展。紫光天化蛋氨酸一期等一批项目建成投产，中电投热电联产、兴尔泰120万吨硝基复合肥等一批项目加快推进，协鑫高效单晶硅等一批战略性新兴产业落地中卫，全年完成工业投资177.2亿元。全市新能源装机容量达3014兆瓦，占全区37.7%，新能源产业产值占工业总产值15.3%。

三、注重生态优先，城乡环境更加美丽

生态环境持续改善。坚持经济建设与生态建设并重，完善生态补偿和修复机制，重点实施了腾格里沙漠防沙治沙、南华山水源涵养林、天然林资源保护、禁牧封育、退耕还林还草等重点生态工程，全市新增造林28.7万亩，森林覆盖率达11.4%。实施黄河湿地恢复保护项目，治理湿地面积近1万公顷。中卫先后荣获“中国生态文明价值城市”、“中国最佳绿色生态城市”。

环保治理成效明显。直面环保问题，召开环保整治誓师大会，全市集中开展了工业企业环保和安全隐患大排查大整治，共排查企业345家，治理环保、安全隐患695个，以钉钉子精神逐一解决环保部挂牌督办的29个突出问题，建立了以“8个体系”和“2个机制”为主的环保治理长效机制。政府投资2.3亿元完成中卫工业园区污水处理厂提标改造和固废填埋场建设，采取PPP模式引入北控集团实施中水回用项目，督促企业投资6.1亿元建设环保治污设施，23家重点企业全部安装在线监测设备，基本实现了废气即时监测、固废安全处理、污水达标排放和水资源循环利用。

统筹城乡步伐加快。实施城市绿化改造提升、亮化、环境和秩序百日整治四大工程，市区水域面积占20%，城市绿地率和绿化覆盖率分别达36.6%和37.9%。全市新建续建城市道路30条44公里，商品房施工面积814万平方米。全国城市环卫保洁工作现场会在中卫召开，232个城市3037人先后来卫参观学习，“以克论净”深度保洁经验全国推广。实施海原大县城建设和中宁旧城改造项目。完成10个特色小城镇和19个“美丽村庄”建设。社区推行“2个10”、农村建立“5个3”深度保洁模式，沙坡头南北干渠及灌区节水改造基本完成，黄河二期防洪中卫段治理进展顺利。集中开展农田水利基本建设和农村环境卫生综合整治，沙坡头区荣获2015年度全区农田水利基本建设“黄河杯”竞赛特等奖，中宁县获一等奖，海原县获二等奖，沙坡头区农田水利建设清工筹劳经验全国推行。

四、围绕民生改善，社会事业全面进步

民生实事全部落实。在财政增速放缓、进入偿贷高峰、支出压力加大的情况下，安排71.5%的财力改善民生。为民承诺12件民生实事全部落实。改造老旧小区65个，新建保障性住房90万平方米，改造棚户区5337户、农村危房5150户。下决心解决了设市以来5.2万被征地农民养老保险遗留问题，下大气力解决了福润苑等拆迁农户逾期3年未安置问题，3832户拆迁农户拿到了钥匙。加快实施中南部城乡饮水安全连通工程，海原1.4万户群众喝上了放心水。新建敬老院3所，建成农村老年幸福院56个，沙坡头区、中宁县城市社区智能化居家养老信息服务实现全覆盖。住房公积金支持11969人改善了居住条件。养老、医疗等覆盖面不断扩大，全区社会保障卡综合应用现场会在中卫召开。实施“1+3”就业创业援助服务行动，城镇登记失业率控制在3.9%以内。

社会事业加快发展。建成宁夏大学中卫校区，整体移交宁夏大学，填补了中卫高等教育空白。中卫市职业技术学校进入国家“中等职业教育改革发展示范学校”行列。沙坡头区、中宁县义务教育均衡发展顺利通过国家评估认定。新建扩建幼儿园14所，学前教育实现全覆盖。创建自治区级科技型中小企业25家，专利申请量166件，授权量64件。建成市人民医院医疗卫生中心，宁夏医科大学总院中宁分院、海原县人民医院迁建工程主体封顶。成功创建自治区卫生城市。全面落实计划生育“三项制度”, 人口素质不断提升。深入实施文化惠民工程，文化演出1127场次，建成一批标准化乡镇文化站和农村书屋，举办中阿博览会篮球四国邀请赛、首届市民运动会等赛事。成功创建自治区文明城市，中卫获全国未成年人思想道德建设工作先进市，5个单位、2个村镇晋升全国文明单位、村镇行列。

扶贫开发扎实推进。全面启动创建海原扶贫开发示范县。发放“双到”资金3040万元，扶持1.5万户建档立卡贫困户发展特色种养业。鼓励企业和民间资本开展产业帮扶，华润集团“基础母牛银行模式”得到汪洋副总理肯定。新建、续建生态移民住房1156套，搬迁安置移民4855人，全面完成“十二五”移民搬迁安置任务。通过整村推进、生态移民搬迁、产业扶贫、社会帮扶，全市34个重点贫困村实现脱贫销号，292个贫困村实现定点帮扶全覆盖，减少贫困人口2.3万人。

社会大局和谐稳定。深化“平安中卫”建设，深入推进网格化管理，加强社会治安防控体系建设，开展打击“两抢一盗”、禁毒人民战争等专项行动，继续保持高压态势，群众安全感、满意度持续提升。强化社会矛盾调处工作，建立覆盖县、乡、村、组四级调处网络，完善“1+X”信访工作机制，全年信访人次同比下降20%。依法加强宗教事务管理，为重点宗教场所委派监督员60名，深入开展和谐寺观教堂创建活动，甘宁两省区宗教工作联席会议在中卫召开。率先推行安全生产第三方监管，全区安全生产责任落实年现场观摩会在中卫召开。扎实推进军民融合深度发展，创建“全国双拥模范城市”通过国家验收。

五、着力深化改革，发展活力不断增强

综合改革全面推进。沙坡头区设区获自治区批复。组建非公有制企业人才发展服务中心，创建宁夏非公有制企业人才发展服务试验区，建成7个人才培养基地和16个人才创新创业实践基地。沙坡头区农村集体经济组织产权制度改革、农村土地承包经营权抵押贷款等5项试点改革顺利推进，中宁工业园区直供电改革正式启动，中宁民营经济综合改革列入自治区改革试点。举办了宁夏投资项目在线审批监管平台专题培训班，搭建了高效便捷的政府投资项目网络平台。

开放合作成效明显。出台加快推进开放中卫建设的实施意见。中国人民大学西部人才培训基地、兰州大学中卫高新技术研究院等挂牌成立，宁夏大学亚马逊云计算学院成为第六轮中美人文交流高层磋商会议成果。成功举办中国枸杞论坛和中阿网上丝绸之路新一代云计算分论坛，陕甘宁蒙毗邻地区共同发展联席会议十二届年会暨第六届经洽会在中卫召开。与31个城市建立旅游协同发展机制，同甘肃白银、内蒙古阿盟等周边地市建立全面战略合作关系，构建全方位、多层次、广领域对外开放新格局。

政务环境不断优化。深化行政审批制度改革，审批事项由229项合并压减为80项，减幅达65%，推行“先照后证”，做到“一站受理、并联审批、限时办结”；取消所有市本级行政审批收费，实行“零收费”；深化商事制度改革，启动运行“三证合一”登记制度，办证时间由15个工作日缩短到3个工作日。全市新登记企业2985家，新增个体工商户8410家，分别增长10.6%和31.3%。

六、突出勤政务实，政府效能明显提升

法治政府不断加强。严格执行 “三重一大”事项集体决策规定。建立市、县政府常务会议学法制度，不断提高政府组成人员的法治观念和依法行政水平。出台依法治市实施意见，完善法治政府建设指标体系，进一步规范政府权力。扎实开展“六五”普法，干部群众逐步向办事依法、遇事找法、解决问题用法、化解矛盾靠法方向转变。自觉接受人大法律监督和政协民主监督，办理人大代表建议42件，当年办结率85.7%，答复率100%；政协委员提案166件，当年办结率90.9%，答复率100%。密切联系工会、共青团、妇联等人民团体，积极支持民主党派、工商联、无党派人士参政议政。

效能政府取得实效。出台《关于提高市政府领导班子行政效率的规定（试行）》，使市政府领导班子成员有职有权有责，职权责对等。召开市政府常务会议21次，决定事关中卫改革发展重大事项248个；召开市长办公会议68次，解决了172个“老大难”问题。市长信箱受理群众来信来电3464件，办结率99.6%。扎实开展“三严三实”专题教育、“从严从实抓落实、大干实干100天”和“二次创业”大讨论活动，深化了思想认识，理清了发展思路，推动了工作落实。实行广场和媒体等多元化问政。制定出台《公职人员不担当慢作为效率低行为问责办法（试行）》，对工作落实不力、作风散漫的16名责任人进行了问责，干部作风明显转变。

廉洁政府扎实推进。始终把纪律和规矩挺在前面，深入开展“清廉中卫”建设，认真落实“一岗双责”。严格执行中央“八项规定”，组织开展明察暗访25次，对5起侵害群众利益案件进行了通报。继续精简文山会海，全年会议、文件分别减少6.7%和12.3%，“三公”经费下降6.3%。深入开展工程建设领域突出问题及机关效能建设专项治理，500万元以下政府投资项目试行“合理造价区间随机抽取中标人办法”评标，公共资源集中阳光交易节约资金2.8亿元。加强国有资产管理，加大审计监督力度。“零容忍”惩治腐败，全年给予党政纪处分157人，挽回经济损失4708.4万元。

一年来，全市国防教育、双拥共建、民兵预备役、防震减灾、人民防空、外事侨务、统计调查、市志档案、邮政通信、气象服务等各项事业都取得了新成绩。

各位代表，总结2015年，我们深感欣慰和不易；回顾“十二五”，我们倍感鼓舞和自豪。5年来，我们经受了前所未有的经济下行压力，办成了一批事关中卫长远发展的大事，办妥了一批制约中卫持续发展的难事，办好了一批促进中卫科学发展的喜事，一个年轻而富有魅力、开放而彰显活力、亮丽而充满实力的中卫屹立在祖国西部！

“十二五”是中卫综合实力大幅提升的5年。地区生产总值连续突破200亿元、300亿元大关，从173.2亿元跃升至316.6亿元，年均增长9.6%，是“十一五”末的1.8倍；累计完成全社会固定资产投资1443.3亿元，是“十一五”的2.9倍；地方公共财政预算收入从8.5亿元增加到21.3亿元，年均增长20.2%，是“十一五”末的2.5倍。

“十二五”是中卫产业转型步伐最快的5年。针对产业结构性矛盾日益突出的复杂局面，我们坚持在发展中调整产业结构、在实践中优化区域结构，初步形成了旅游、信息、现代物流、现代农业和新型工业化“四主一化”产业支撑体系，发展质量和效益明显提升。三次产业结构由19:40.8:40.2调整为16.5:44.6:38.9。

“十二五”是中卫城乡面貌变化最大的5年。深入实施城市建设“五大战役”和城市“六创”，先后建成“大河之舞”主题文化公园、香山公园、黄河公园、应理湖、五馆一中心、腾格里湖、“十里水街”等地标性建筑和市民休闲公园，市区建成区面积由23平方公里扩展到32平方公里，中卫先后荣获“国家园林城市”、“全国十佳生态文明建设示范城市”，“宜居、休闲、生态美”成为中卫亮丽名片。

“十二五”是中卫人民得到实惠最多的5年。累计落实民生实事56件，建设各类保障性住房6.5万套，搬迁生态移民9218户3.9万人，减贫10.4万人，一批事关群众切身利益的就学、看病、饮水、居住、出行等难题得到有效解决。中卫先后荣获全国六星级、七星级慈善城市。城镇常住居民人均可支配收入从13980元提高到21604元，年均增长10.7%，是“十一五”末的1.5倍；农村常住居民人均可支配收入从4439元提高到8002元，年均增长12.2%，是“十一五”末的1.8倍。

看似寻常最奇崛，成如容易却艰辛。各位代表，回首这5年，我们感念党中央、国务院和自治区党委、政府的亲切关怀与厚爱，感谢社会各界的大力帮助与支持，感激全市113万回汉各族人民的卓越创造与无私奉献。在此，我代表市人民政府，向辛勤耕耘在各条战线的广大干部群众，向给予政府工作大力支持的人大代表、政协委员、各民主党派、工商联和各界人士，向中央、区属驻卫单位、驻卫部队、武警官兵，致以崇高的敬意！向所有关心支持中卫发展的港澳同胞、台湾同胞、海外侨胞和各界朋友，表示衷心的感谢！

在肯定成绩的同时，我们也清醒地看到，面对新常态，我市经济社会发展还存在一些亟待解决的问题，主要表现在：一是发展不足仍是最大市情，经济总量小，人均地区生产总值仅为全区63%、全国56.5%；二是创新驱动发展能力不强，创新人才短缺，改革的“硬骨头”比较多，创新创造的体制机制还不完善；三是山川城乡发展不平衡，基本公共服务供给不足，贫困人口基数大，脱贫攻坚任务重，城镇、农村居民收入水平与全面建成小康社会目标还有较大差距；四是依法行政意识和能力有待增强，新常态下有些干部为政不为、慢作为、不担当的问题较为突出，政府效能还需进一步提高。对于这些问题，我们必须高度重视，采取有力措施，切实加以解决。

“十三五”发展目标任务

“十三五”时期，是全面建成小康社会的决胜期，也是中卫改革发展的攻坚期，更是开放创新转型追赶的机遇期。今后5年，政府工作的总体要求是：高举中国特色社会主义伟大旗帜，全面贯彻党的十八大和十八届三中、四中、五中全会精神，以马克思列宁主义、毛泽东思想、邓小平理论、“三个代表”重要思想、科学发展观为指导，深入贯彻习近平总书记系列重要讲话精神，按照“五位一体”总体布局和“四个全面”战略布局，围绕市委三届六次全体会议部署，以五大发展理念统领开放富裕和谐美丽中卫建设，以开放创新、转型追赶为主线，以提高发展质量和效益为中心，坚持市域统筹、县区协同、产城融合、城乡一体、山川共济，大力实施“开放引领、创新驱动、富民共享、生态优先”战略，全力打造全域旅游城市、国际云端城市、特色产业城市、物流枢纽城市、生态宜居城市，坚决打赢脱贫攻坚战，与全国全区同步全面建成小康社会。

主要预期目标是：经济保持中高速增长，地区生产总值年均增长8%左右，地方公共财政预算收入与经济增长同步，城镇、农村常住居民人均收入年均分别增长9%和10%左右，力争实现“一个翻番、两个提前、三个缩小、五个全面提升”目标。“一个翻番”，即2018年地区生产总值在2010年基础上实现翻番；“两个提前”，即2018年全市贫困人口全部提前脱贫，海原贫困县提前摘帽；“三个缩小”，即城镇、农村居民收入和城镇化率三项指标与全区平均水平的差距明显缩小；“五个全面提升”，即基本公共服务均等化水平全面提升，全民思想道德素质全面提升，社会治理能力全面提升，生态环境质量全面提升，政府效能全面提升。

工作措施是：实施四大战略，建设五个城市，打赢脱贫攻坚战，抓好66个重大项目。

实施四大战略。一是实施开放引领战略。中卫是内陆欠发达地区，目前我们面临的最大问题仍然是发展不足、速度不快、质量不高，制约发展的最大瓶颈是思想观念保守、开放意识不强、创新能力不足。“十三五”时期，我们要深度融入国家“一带一路”建设，充分借助宁夏内陆开放型经济试验区和中阿博览会两块“金字招牌”,积极构建大开放格局，加快建设网上丝绸之路宁夏枢纽工程，以“五大理念”统领“四个中卫”建设，以改革开放引领“十三五”发展，实现中卫大发展、快发展。

二是实施创新驱动战略。创新的核心要素是科技创新，我市科技创新投入不足，企业自主创新能力严重不足，成为我市转型发展的重要制约因素。“十三五”时期，我们要从投资驱动、要素驱动为主向创新驱动为主转变，进一步加大创新投入，结合我市云计算、旅游、现代农业、现代物流、新型工业等主导产业，实施一批重大科技专项，培育一批专精特新和科技型中小企业。大力实施育才、引才工程，探索建立“政策+项目+人才”的新机制，有效破解人才短缺瓶颈，以人才创新引领科技创新，以科技创新提升发展质量和效益。

三是实施富民共享战略。小康不小康，关键看老乡。我市城乡、山川、区域发展不平衡，海原县集中了全市70%的贫困人口，人均地区生产总值仅为全区、全市平均水平的23.1%和36.7%，小康社会实现程度全区最低；我市城镇居民人均可支配收入是农村居民的2.7倍，且呈拉大趋势。“十三五”时期，我们必须坚持共享发展理念，牢固树立全市“一盘棋”思想，坚持走市域统筹、县区协同、城乡一体、山川共济的发展路子，在统筹协同上做文章，在补齐短板上下功夫。要在加快海原脱贫攻坚的同时，充分发挥卫宁平原引黄灌溉、区位交通、发展基础等综合优势，打造中卫经济新的增长极，引领全市加快发展。

四是实施生态优先战略。吸取腾格里沙漠污染事件深刻教训，牢固树立绿水青山就是金山银山的理念，把生态文明建设融入经济社会发展全过程，更加自觉地推动绿色发展、循环发展、低碳发展，坚持土地、资源和环境底线，全力打造生态宜居城市，走生产发展、生活富裕、生态良好的发展之路。通过健全科学合理的生态保护与补偿机制，严守基本农田、水资源红线，加大环境治理和生态保护，构筑科学合理的城镇化格局、农业发展格局和生态安全格局，努力使生态美、城乡美、和谐美与产业强、百姓富、保障好的目标高度契合。

建设五个城市。一是建设全域旅游城市。充分发挥中卫独特的旅游资源优势，坚持优先发展旅游业，实施旅游“五项行动”，构建全域旅游空间格局，推动旅游与城市、文化、其他产业高度融合，力争全市接待游客1100万人次，旅游总收入超过120亿元，占地区生产总值比重达到15%以上，将旅游业发展成富民产业和幸福产业，在全区率先创建国家全域旅游示范市，继而把中卫打造成为宜居宜游的国际性全域旅游目的地、国际化沙漠旅游目的地城市和中国西部独具特色的旅游休闲度假城市。

二是建设国际云端城市。以云计算为主的“互联网+”产业是转型升级的最大优势和最强动力，抢抓国家“信息丝路”计划实施和建设网上丝绸之路宁夏枢纽机遇，坚持国际化视野、专业化团队、市场化运作的理念，以建设“前店后厂”新一代云计算基地和国家战略数据安全灾备基地为目标，充分发挥亚马逊AWS的龙头带动作用，加快建设西部云基地，推动军民融合产业发展，着力打造政策洼地，完善产业链条，培育竞争优势，把西部云基地打造成为中阿信息港和网上丝绸之路宁夏枢纽。力争服务器规模达到100万台，年服务收入突破300亿元。

三是建设特色产业城市。深入推进工业园区提质改造等七项行动计划，加大冶金、新材料、精细化工等传统产业技术改造升级，下决心淘汰碳化硅、小冶金、机制纸等技术装备水平低、缺乏市场竞争力的落后产能，重点发展新能源、新材料、环保等战略性新兴产业，集中力量打造4个百亿元、1个千亿元产业集群，力争工业总产值突破1000亿元，工业对经济增长的贡献率达40%左右。坚持“一特三高”，突出发展枸杞、硒砂瓜、草畜、马铃薯、果蔬等优势产业，着力构建“四区七带”农业产业布局和现代农业体系；突出特色化、品牌化、精品化，加强农业自主创新，培育示范、龙头企业，推动农业由生产导向向消费导向转变，实现农业增产、农民增收。力争农业增加值达到63.2亿元。

四是建设物流枢纽城市。积极融入“一带一路”，全力构建“网上、空中、陆上”三位一体的辐射西部、直通全国、连接中东的便捷通道。网上，实施“互联网+”行动计划，加快建设宁夏中关村科技产业园西部云基地和中卫云中心，创建智慧城市，构筑云设施、云应用、云服务产业链，让云计算像用水、用电一样，进入千家万户。空中，实施沙坡头机场改造提升工程，加密固化中卫至北京等大城市航线，将沙坡头机场建成银川河东国际机场的备降机场和国家低空开放的试验机场。陆上，加快建设四通八达的对外快捷通道，加快构建综合物流网络，使区位交通优势转化为产业发展优势。力争全社会物流总额突破1000亿元。

五是建设生态宜居城市。统筹城乡发展，按照“三规合一”“多规融合”，完成城乡总体规划，重点区域控制性详规覆盖率达到100%。围绕“一核三轴四区”城镇发展布局，通过“产业连城、生态连城、水系连城、交通连城”，加快推进沙坡头区与中宁县相向发展。按照“城市郊区村落向市区集中、城镇周边村落向集镇集中、分散村落向中心村集中”的思路，加快推进城镇化进程，全市城镇化率达到52%。实施腾格里沙漠防沙治沙、南华山水源涵养林、生态移民迁出区生态修复、农田林网、城乡绿化等重点生态工程，开展蓝天、绿水、净土三项行动，做到源头严防、过程严管、后果严惩，为子孙后代保护好蓝天白云、绿水青山。到2020年，全市森林覆盖率达到14%，城市绿地率达到44%。

打赢脱贫攻坚战。以海原县为主战场，认真落实中央“六个精准”“五个一批”和自治区党委书记李建华“五个走在前”要求，举全市之力打赢打好脱贫攻坚战。重点做好“有土”扶贫和“离土”扶贫两篇文章，形成“内外合力”。在“有土”上下真功，争取实施西线扶贫供水工程，帮助中南部群众摆脱靠天吃饭困境；在“离土”上出实招，鼓励农民离土创业，脱贫致富，生活得更有尊严。“对内”通过精准发展特色产业、转移就业、生态移民、教育扶贫等，推动脱贫一批；“对外”积极争取国家、自治区和社会力量支持，鼓励民营企业参与，带动脱贫一批，确保2018年现行标准下11.8万贫困人口全部脱贫，152个贫困村全部脱贫销号，扶贫开发工作走在全区前列。

抓好66个重大项目。科技创新方面，重点抓好宁夏中关村科技产业园中卫云中心、宁夏枸杞核心产业带等4个项目，力争完成投资85.2亿元。现代农业方面，重点抓好宁夏中部干旱贫困片区西线供水工程、光伏高效生态农业科技示范园等10个项目，力争完成投资80.2亿元。工业方面，重点抓好协鑫10GW单晶、华电海原新能源发电等12个项目，力争完成投资890亿元。服务业方面，重点抓好沙坡头旅游新镇、中国物流中卫物流园等8个项目，力争完成投资37.4亿元。信息化方面，重点抓好亚马逊AWS、中国联通数据中心等5个项目，力争完成投资140.2亿元。新型城镇化方面，重点抓好市区地下综合管廊、棚户区改造等4个项目，力争完成投资84.8亿元。社会民生和生态保护方面，重点抓好生态移民、环城森林公园等6个项目，力争完成投资60.4亿元。基础设施方面，重点抓好中卫至兰州客运专线、太阳山至海原铁路等17个项目，力争完成投资1055.3亿元。

2016年主要工作

2016年是“十三五”开局之年，是全面建成小康社会进入决胜阶段的第一年，也是推进供给侧结构性改革的攻坚之年。今年的主要预期目标是：地区生产总值增长8%左右；全社会固定资产投资增长12%；地方一般公共预算收入与经济增长同步；社会消费品零售总额增长9%；城镇和农村常住居民人均可支配收入分别增长9%和10%；居民消费价格指数控制在3%左右；城镇登记失业率控制在4.5%以内；节能减排完成自治区下达任务。

按照市委的安排部署，重点抓好以下五项工作：

一、坚持改革创新，加快建设开放中卫

始终把改革创新贯穿经济社会发展全领域，依靠改革创新挖掘潜力、培育优势、拓展空间、激发活力、推动发展。

突出抓好结构性改革。全面落实国家和自治区去产能、去库存、去杠杆、降成本、补短板政策措施，推动经济平稳较快发展。去产能方面，调控钢铁、水泥、电解铝、电石发展，重点淘汰鸿森矿12500KVA铁合金矿热炉、天净天能5万吨碳化硅等一批落后产能。去库存方面，鼓励市内各类建设项目优先使用钢铁、水泥、瓷质砖、铝型材等大宗商品库存；尚未分配的1768套15万平方米棚户区安置房，3月底前全部按程序分配到位；空置的8331套93.2万平方米商品住房，放宽购置条件，加大促销力度，释放改善性住房需求。去杠杆方面，重点做好防范非法集资、资金链断裂、联保互保代偿风险、新兴金融业态潜在风险。降成本方面，专项清理涉企收费，降低职工“五险一金”缴费比例，减免过路过桥费用，降低企业用工、用电等成本。补短板方面，不断完善水、路等基础设施，补齐环境保护、安全生产、土地利用等方面短板，为实现长远发展、可持续发展奠定基础。

加快推进重点领域改革。进一步理顺沙坡头旅游经济开发试验区、中卫工业园区管理体制机制，完善各类服务职能。大幅精简规范行政审批事项，建立投资项目网上在线审批监管平台，计划6月底前向社会公布行政审批“五个清单”。组建市行政审批服务局，加快建设“政务云”平台，实行网上审批、网上注册、网上办事，实现行政审批“一张网”全覆盖。3月底前完成公务用车制度改革。实行集体所有权、农户承包权、土地经营权“三权分置”，在沙坡头区开展农村承包土地经营权抵押贷款试点。强化土地储备职能，推进城乡建设用地增减挂钩试点改革。积极推进水资源使用权确权登记，抓好水权交易试点工作。深化文化体制改革，推进“公建民营公助”模式。加快医药卫生体制改革，推动县级公立医院综合改革。深化登记制度改革，探索推行“证照分离”登记模式。推进中宁工业园区供电体制改革试点，加快不动产统一登记发证工作。

不断创新财税金融机制。做强做实金融工作办公室引领、协调与服务职能，促进产融结合。发挥财政资金杠杆作用，扩大地方经济发展过桥转贷、主导产业发展和农业产业发展贴息、小微企业贷款保证保险等“资金池”规模。引导社会资本参与组建中卫市担保公司，建立“先担保、再考核、后奖补”的“政银担”合作模式。深化国地税征管体制改革，落实结构性减税政策。完善金融服务体系，支持各类金融机构设立分支机构。引进私募股权基金，鼓励符合条件的企业组建财务公司、金融租赁公司。创新投融资体制，依托资本市场和债券市场，加快推进直接融资；积极开展互联网金融业务，加强与保险公司、证券公司等合作，探索成立中卫市交通物流投资集团，推广政府与社会资本合作（PPP）模式，撬动更多社会资本参与重大项目和基础设施建设。

全面扩大对外开放。贯彻落实《关于加快推进开放中卫建设的实施意见》，实行全方位、多层次、广领域开放。加强与北京、重庆、江苏、福建、山东等发达地区的战略合作，密切与宁蒙陕甘毗邻城市的务实合作，构筑对外开放新格局。积极争取自治区对外开放产业基金，鼓励企业采取并购、风险投资等方式，拓宽投资合作渠道。不断深化医院、学校与区内外优质资源合作。出台《中卫市招商引资扶持激励政策》，实施“8513”招商行动，开展民营企业中卫行活动，突出以商招商、专业招商、委托第三方招商，开展建链、补链、延链、强链集群式招商，力争全年走访城市50个、企业100家，对接落实投资亿元以上项目30个，完成招商引资到位资金300亿元。

二、坚持转型升级，加快建设富裕中卫

实施工业强基、农业提质、信息壮大、旅游升级“四大工程”，抓好60个重点项目建设，以项目增投资、以项目促转型，不断提高发展质量和效益。

强力推进新型工业。牢固树立工业强市理念，坚持园区发展与产业培育并重，大企业与中小微企业并存，扩增量与调存量并举，提升工业发展质量。积极发展光伏、风电，年内建成新能源装机容量750MW；大力发展新能源制造业，抓好京能2×660MW热电厂等6个项目；培育发展节能环保产业，抓好中卫危废处理中心等9个项目；改造提升冶金、新材料产业，抓好锦宁巨科20万吨高精铝板带箔深加工等7个项目；坚持高新技术、高附加值、高度安全和生态环保优先的发展方向，抓好兴尔泰120万吨硝基复合肥等7个项目；开工建设中卫农产品加工园区，围绕新水等特色农产品加工园区，抓好天士力枸杞深加工等12个项目。实施工业园区基础设施提升和环境综合整治工程，推进中卫、中宁工业园区一体化发展。确保规模以上工业增加值增长10%以上。

稳步发展现代农业。围绕“一特三高”，大力推进中卫国家农业科技园区和国家现代农业示范园区建设，做优做强优质粮食+枸杞、硒砂瓜、草畜、马铃薯、果蔬“1+5”优势特色产业，实施苹果产业扩规提质增效树品牌工程，创建粮食高产示范区11个，建设优质水稻生产基地12万亩、蔬菜标准化园区8个，新增枸杞3万亩、农作物制种1.5万亩，建成硒砂瓜品质品牌保护核心区40万亩，新建千只肉羊标准化养殖场33个、千头肉牛养殖场8个。建成国家级枸杞质量检测中心。实施“互联网+农业”行动计划，新培育市级以上示范合作社15家、家庭农场15家、农业龙头企业5家。积极推进宁夏中部干旱贫困片区西线供水工程前期工作，加快建设黄河城市过境段综合治理及灌区节水续建配套工程，新增高效节水灌溉8万亩。开展现代农业暨特色优质农产品“六个一”宣传活动。认真搞好第三次全国农业普查，全面摸清“三农”新家底。力争全年农业增加值增长5%以上。

加快发展信息产业。加快西部云基地配套基础设施和网络通道建设，建成中卫—北京、中卫—西安4×100G骨干网络通道，力促亚马逊AWS、奇虎360等数据中心项目一期上线运营。全力推进中国移动、中国联通数据中心等6个云计算项目建设。加强对接，推动军民融合、游戏影视、气象服务、卫星遥感等灾备中心项目落户中卫，打造国家战略数据安全灾备基地，力争西部云基地服务器装机容量达到10万台。积极发展云制造产业，争取美国ZT等服务器组装生产项目落地。落实高速宽带网络建设和提速降费行动方案，推进“宽带中国”示范城市建设，年内建成通讯基站230个，沙坡头区、中宁县光网通村入户率达38%，海原县宽带网络实现“乡乡通”。加快建设宁夏中关村科技产业园中卫云中心和云应用项目建设，培育“两化”融合试点企业5家，着力构建云制造、云应用、云服务核心产业链，打造网上丝绸之路宁夏枢纽工程。

大力发展全域旅游。编制完成《中卫市全域旅游发展行动计划（2016-2018）》，全力创建国家全域旅游示范城市。实施沙坡头旅游景区和腾格里沙漠湿地提质扩容、中国枸杞博览中心、海原回乡民俗一条街等27个重点项目，年内完成投资48亿元。加快旅游新镇核心区建设，5月1日前封闭运行。加大高墩湖养生养老产业园、腾格里湿地公园沙漠温泉、银阳光伏科技生态园及海原天都山等项目招商。督促港中旅加大投资。加强与陕旅集团等合作，把中卫变成银川、西安、兰州的“后花园”。不断完善吸引物系统、交通组织系统和综合配套服务系统，开通直达各景区的旅游公交线路，完善景区交通指引标识引导服务。实施“十百千万”工程，加强旅游市场秩序整治。加快推进 “旅游云”平台建设，成立中卫市旅游营销中心，加强与途家、同程等国内知名旅游电商深度合作。积极发展乡村旅游。做大做强大漠黄河旅游节等旅游节庆，提升中卫旅游知名度和美誉度。力争全年游客人数和旅游总收入均增长28%。

积极发展交通物流。以打造丝绸之路经济带交通物流枢纽城市为目标，全力推进吴忠至中卫城际铁路、中卫至兰州客运专线、中卫高铁站及跨黄河公路桥、乌玛高速中卫段、海同高速公路等项目建设，在中卫对外重大通道建设上取得突破。加快中国物流中卫物流园、中宁星火国际物流园、镇罗公铁物流中心等物流园建设，力争年内入园企业70家，实现物流业破题发展。开通中卫至中亚国际专列，推动中宁陆路口岸封关运营。开通加密中卫至北京、西安等城市航线航班。建立中卫物流运价指数，打造云物流信息港。加快农村公路建设。大力发展电子商务、供应链物流、金融物流等，打造区域现代物流中心。做足“物流+旅游”“物流+商贸”“物流+金融”3篇文章，将中卫打造成为中国重要的云物流信息港和丝绸之路经济带上的重要物流节点城市，加快构筑中阿陆上丝绸之路区域枢纽。

三、坚持民生为要，加快建设和谐中卫

始终把群众的冷暖放在心中、抓在手上、落到实处，全力办好15件民生实事，让全市人民共享改革发展成果。

全面发展社会事业。把加快教育发展摆在更加突出的位置，整合教育资源，提高教育质量。加快发展学前教育，扩大市第二、第三幼儿园等办学规模；实施薄弱学校改造及教育信息化全覆盖工程，新建、改扩建中小学18所7.1万平方米，校校通、班班通、人人通实现率分别达91%、76%、80%；推广“小班化”教学模式，推进义务教育均衡发展；创新职业教育发展模式，健全完善中高职贯通教育和校企互联互聘机制；支持宁夏大学中卫校区建设，配合办好亚马逊云计算学院。办好第31届宁夏青少年科技创新大赛等活动，年内建成各类科技基础创新平台10家，建立众创空间2个。加快建设市人民医院医技楼、市疾病预防控制中心，扩建3所乡镇卫生院，持续开展城乡居民普惠性健康体检。落实一对夫妇可生育两个孩子政策。加快建设黄河宫体育公园、中宁县城市文化中心、海原县非物质文化遗产传承基地，扎实推进文化惠民工程，积极培育新兴文化业态。办好第三届全国大漠体育健身运动大赛、环青海湖国际公路自行车赛等知名赛事。加大对农村留守老人妇女儿童关爱服务，推进残疾人事业健康发展。

大力推进精准扶贫。召开全市脱贫攻坚誓师大会，制订《中卫市三年打赢脱贫攻坚战实施方案》，出台贫困群众“离土”脱贫若干扶持政策，以打造海原扶贫开发示范县为重点，搭建扶贫云大数据平台，实行有进有出动态管理，按照“六个精准”要求，实施“六个一批”措施，深入推进“四到”工程，建立健全“六项机制”，做好“有土”和“离土”两篇文章，切实做到精准扶贫、精准脱贫，全年减少贫困人口3.4万人。实施“金扶工程”，启动建设4097户易地扶贫安置项目。整合资金4亿元，实施34个整村推进重点贫困村脱贫销号。争取“双到”资金3010万元，扶持1.5万户建档立卡贫困户发展特色种养业。发放千村信贷互助资金项目捆绑贷款8000万元以上。大力发展光伏扶贫。推进沙坡头区移民村资产收益扶贫试点，盘活土地资源。落实贫困家庭大学生资助计划。加大贫困人口职业技能培训，引导贫困人口有序转移。整合社会资源，形成脱贫合力。从今年开始，全市行政公用经费再压缩5%，全部用于脱贫攻坚工作。

完善社会保障体系。实施全民参保登记计划，加快建立“五险合一”经办管理体制。全面启动机关事业单位工作人员养老保险制度改革，积极落实被征地农民参加养老保险和事业单位工作人员医疗补助政策。健全城乡社会救助体系，落实城乡居民大病保险制度。拓展社会保障卡综合应用功能，稳步推进跨省区基本医疗费用异地即时结算。全市建设（或购买）安置住房1.2万套110万平方米，改造老旧小区66个，实施既有居住建筑节能改造55万平方米。加快1.7万套91万平方米公租房建设，逐步扩大公租房保障范围。规划建设市养老产业园，建成市、县老年人活动中心、市关爱护理院和5所敬老院。实施更加积极的就业创业政策，加强人才队伍建设，打造大众创业、万众创新和增加公共产品、公共服务“双引擎”。

创新社会治理方式。深入推进平安中卫创建，实施“互联网+社会治安”行动计划，完善群众诉求表达、矛盾调处、权益保障机制和涉法、涉诉信访依法终结制度, 推广社会治安网格化管理，力争刑事发案率控制在5％。以内。启动“七五”普法，扎实推进“法律八进”活动。建立健全“四级五覆盖”安全生产责任体系，坚决防范重特大安全事故发生。加强食品药品监管，保障舌尖上的安全。做好应急储备和防灾减灾工作，提高突发事件的应急处置能力。依法管理宗教事务，不断巩固民族和睦、宗教和顺、社会和谐的良好局面。

四、坚持统筹协调，加快建设美丽中卫

优美环境是中卫最大优势和亮丽名片，我们要像爱护自己的眼睛一样，倍加珍惜和保护环境，让生态宜居世代相传，让“中卫蓝”永驻。

打造美丽城市。开工建设高庙历史文化街区，对“三片区五街道”100栋建筑外立面进行仿古改造。实施城市热电联网配套工程、城市污水处理厂提标改造工程，新建、改造城市道路及街巷29条31公里，新建续建观河大院、金岸榕苑等房地产项目，加大城市核心区改造力度，逐步消除城市规划区“城中村”，不断提升城市功能。实施滨河路改造，建设沿黄河景观带，推进沙坡头区与中宁县城同城化发展。继续推进海原大县城建设。建设“城市环卫云”系统，全面运营数字化城市管理系统和静态交通系统，深入推进“以克论净”深度保洁模式，启动创建国家卫生城市。实施城市管理相对集中行政处罚权，提升城市管理水平。深入推进全国文明城市和国家环保模范城市创建工作。实施沿卫宁黄河段绿化景观带、市区环城森林公园二期、城市湿地森林公园、中宁丝路方舟森林公园、海原牌路山绿化三期、海兴开发区苋麻河森林公园和凤凰山森林公园等项目，全市城市绿化覆盖率达到38%。加快推进户籍制度改革，吸引更多人口向市区聚集，努力打造生态宜居城市。

建设美丽乡村。中卫美，乡村必须美。抓好常乐、三河等5个特色小城镇和姚滩、白马等19个美丽村庄建设，改造农村危房3500户。开展“农村环境卫生整治年”活动，将农村环境综合整治与产业培育相结合，探索推行“城市郊区村落向市区集中、城镇周边村落向集镇集中、分散村落向中心村集中”的村镇发展模式，全面推进农村“5个3”保洁机制，切实改善农村面貌。抓好主干道、沿山沿河林带林网建设，推进湖泊湿地保护、移民迁出区生态修复、北部沙区综合治理、南华山水源涵养等生态防护体系建设，巩固扩大封山禁牧、退耕还林还草成果，完成造林绿化面积27.6万亩。坚守耕地红线，确保占补平衡耕地质量，全市耕地保有量保持在393万亩，基本农田巩固在292万亩。

保护美丽环境。全面建成中卫工业园区污水处理厂中水回用项目，确保环保部挂牌督办事项全部摘牌。加强工业大气污染治理，督促电石、铁合金等行业企业建成余热发电项目，完善企业治污、除尘、脱硫、脱硝设施。实施节能降耗行动计划，加强重点用能企业能源管控，落实差异化用能政策。加快推进城市集中供热，全面淘汰城市建成区燃煤锅炉，加强城市生活源大气污染治理。实施生活垃圾焚烧发电和餐厨废弃物资源化利用、无害化处理。加强城乡集中式饮用水源地保护，加大农村面源污染治理。建立健全查处环境违法行为联动机制，推行环境保护网格化管理，努力营造天蓝、地绿、水清、气爽的生态环境。

五、坚持依法行政，加快建设法治政府

牢固树立法治理念，全面推行依法行政，努力打造职能科学、权责法定、执法严明、公开公正、廉洁高效、守法诚信的法治政府。

行政决策科学化。加强法治政府建设，依法设定权力、行使权力、制约权力、监督权力。与人大对接，在城乡建设、环境保护、文化保护等方面加快立法，做到依法行政。继续执行政府常务会议学法制度，把公众参与、专家论证、风险评估、合法性审查、集体讨论决定作为政府重大行政决策的法定程序，确保决策科学、民主、合法。在作出重大决策时，主动向市委、人大报告，与政协沟通。建立重大决策合法性审查、重大决策终身责任追究及责任倒查制度，坚持有错必纠、有责必查，切实提高政府领导班子科学执政的能力和水平。

监督制约公开化。自觉接受人大及其常委会的法律监督和政协的民主监督，加快推进协商民主制度化建设，高度重视社会公众和舆论监督，充分发挥工会、共青团、妇联等人民团体的重要作用。完善政府内部层级监督，加大对执法部门的检查和监督，细化、量化行政裁量标准，落实执法人员资格管理和行政执法责任制，切实规范执法行为。加大政务公开，及时准确向社会公布财政预算、公共资源配置、重大项目实施、社会公益事业发展等重点信息，主动接受人民群众监督。

作风建设常态化。深化“三严三实”专题教育成果，坚持把纪律挺在前面，驰而不息纠治“四风”。加大执法检查、廉政监察和效能监察，着力解决群众身边的不正之风和腐败问题，持续保持遏制腐败高压态势。深化电视、报纸、广播、网络、微博、微信“六位一体”媒体问政机制。强化领导干部经济责任审计和行政问责，防止权力失控、决策失误、行为失范。强化政府工作目标任务的考核和督察，做到监督与落实并行、问责与问效同步。围绕新常态怎么看、怎么干，调适发展理念，调适思维方式，调适职能定位，调适工作方法，调适作风状态，真正使干部把推动发展的责任扛在肩上、把心思用在工作上、把功夫下在落实上，用勤奋敬业的工作、满腔热情的服务、廉洁奉公的品德和务实苦干的实际行动，树立政府威信，赢得人民信任。

各位代表，小康大业，实干为基；宏伟蓝图，落实为要。新的目标承载新的梦想，新的征程充满新的希望。让我们在自治区党委、政府和市委的坚强领导下，以实打实的作风干工作，以钉钉子的精神抓落实，提振精气神，汇聚正能量，奋力推进“十三五”良好开局，加快建设开放富裕和谐美丽中卫，为全面建成小康社会而不懈奋斗！
